# Single molecule dynamics in a virtual cell: a three-dimensional model that produces simulated fluorescence video-imaging data

**DOI:** 10.1098/rsif.2014.0442

**Published:** 2014-09-06

**Authors:** Gregory I. Mashanov

**Affiliations:** Division of Physical Biochemistry, MRC National Institute for Medical Research, Mill Hill, London NW7 1AA, UK

**Keywords:** single molecule imaging, cell model, object-oriented modelling

## Abstract

The analysis of single molecule imaging experiments is complicated by the stochastic nature of single molecule events, by instrument noise and by the limited information which can be gathered about any individual molecule observed. Consequently, it is important to cross check experimental results using a model simulating single molecule dynamics (e.g. movements and binding events) in a virtual cell-like environment. The output of such a model should match the real data format allowing researchers to compare simulated results with the real experiments. The proposed model exploits the advantages of ‘object-oriented’ computing. First of all, the ability to create and manipulate a number of classes, each containing an arbitrary number of single molecule objects. These classes may include objects moving within the ‘cytoplasm’; objects moving at the ‘plasma membrane’; and static objects located inside the ‘body’. The objects of a given class can interact with each other and/or with the objects of other classes according to their physical and chemical properties. Each model run generates a sequence of images, each containing summed images of all fluorescent objects emitting light under given illumination conditions with realistic levels of noise and emission fluctuations. The model accurately reproduces reported single molecule experiments and predicts the outcome of future experiments.

## 1. Introduction

The past two decades have been marked by the rapid progress in single molecule imaging in live cells. In the early studies, small particles, specifically attached to membrane molecules, were used to track movements of individual molecules [1–3]. Specific fluorescent probes, containing tens of molecules, were also used for similar purposes [4,5]. Progress in camera technology, optics and lasers made it possible to detect and track single membrane-associated molecules tagged with a single fluorophore [6,7] or fused to a single fluorescent protein [8,9] which could be reliably identified as the single molecule of interest. Fast sensitive cameras have allowed detection and tacking of large protein complexes moving within the cytoplasm and at the cell nucleus [10,11]. The application of total internal reflection fluorescence microscopy (TIRFM) has allowed researchers to illuminate a very thin layer of the solution close to the surface of the glass–water interface which greatly improves the signal-to-noise ratio [12], critical for single molecule detection. Some intracellular proteins containing membrane binding domains were detected and tracked at the plasma membrane, because molecules slow down and become visible as discrete spots of light upon binding to the membrane [6,13–15]. Some fine details of molecule movements, which include confined diffusion [2,16], presence of membrane barriers [17] or membrane microdomains [7,18], were evaluated using single molecule or single particle-tracking methods.

One of the most important issues in single molecule research is the stochastic nature of single molecule events, which require large datasets to be analysed in order to obtain statistically valid conclusions. This problem can be solved by using automatic detection and tracking algorithms [5,19] that eliminate operator bias associated with the laborious manual processing of data.

Another important issue is a requirement to cross-validate the results obtained by automated analysis procedures. This problem can be addressed by using computer models to simulate the mobility and binding kinetics of the molecules of interest and to generate the results in the same format as real data. Many of the published works contain a modelling section [10,11,17,19–21] simulating experimental results. Naturally, these models are restricted to a particular problem and are limited in structure and scope. Andrews & Bray [22] proposed a general model emulating chemical interactions between individual molecules of one type moving inside a virtual bacterial cell. This model was modified by Tournier *et al.* [23], who proposed calculating the probability of interaction between two molecules separated by a significant distance, so that the model could use much longer time steps than the Andrews–Bray model. The models can produce results in the form of spatial distributions of individual objects in a particular chemical state and as a time course of the concentrations of the reacting species. These models use only one type of molecule (free moving in cytoplasm), require a significant number of calculations, and do not simulate imaging conditions affecting the results of real experiments.

The purpose of this work was to construct a novel computer model that can simulate a few distinct ‘classes’ of single molecules moving both within the cytoplasm and at the cell membrane and simulate chemical interactions between molecules of the same or few different classes. The model takes into account the three-dimensional illumination pattern created under given conditions (epi-illumination, TIRFM, confocal microscopy) which would affect the emission rate of individual molecules. The images of all the light-emitting molecules, within and beyond the focal plane, are projected onto a virtual imaging device (e.g. EMCCD camera) using rules of optics and experimentally or empirically determined noise and signal characteristics. The model is optimized to use the minimal number of calculations and has a modular structure giving it the ability to be extended to more complex scenarios such as abnormal diffusion, directed movements and single molecule dynamics in the presence of some intracellular structures (e.g. nucleus or cytoskeleton), and to add any new properties (e.g. take into account the effects of polarized illumination and the orientation of the molecules).

The proposed model was extensively tested under a number of scenarios. The results of the modelling (analysed with the use of the automatic detection and tracking algorithms [19]) accurately reproduce the results of the reported experiments [13,14,24–28]. The model was also used to evaluate the putative effects of partial permeability of membrane barriers and of the viscosity of lipid rafts on the outcome of single molecule experiments.

## 2. Model

The basic model may contain objects of one or two major classes: cytoplasm-based molecules moving inside an enclosed volume (‘cell’), and/or objects moving on the surface of this volume (‘plasma membrane’). An object of either class has a set of default properties including physical coordinates (*x, y, z*); mobility coefficient; indexes for a bound object(s) of the same or other classes; up to eight fluorescent tags; a colour of fluorescent tag (if required). Intracellular ‘molecules’ can move freely inside a ‘cell’, and (if permitted) bind to the ‘molecules’ belonging to ‘membrane-based’ classes. The ‘bound-to-membrane’ intracellular molecule moves together with its membrane-embedded ‘partner’ until the moment of dissociation. It is assumed that the mobility of such a pair is equal to the mobility of the slow-moving membrane-based molecule because it is embedded in a viscous membrane. If two membrane-embedded molecules with mobility *D*_a_ and *D*_b_ bind each other, the mobility of the pair (*D*_pair_) will be proportionally slower than the mobility of each member of the pair: *D*_pair_ = (*D*_a_)/(1 + *D*_a_/*D*_b_). For example, if two membrane molecules form a homodimer (see §2 in the electronic supplementary material), then its mobility will be two times slower than the mobility of the monomer (see table S4 in [24]).

**Figure 1.**
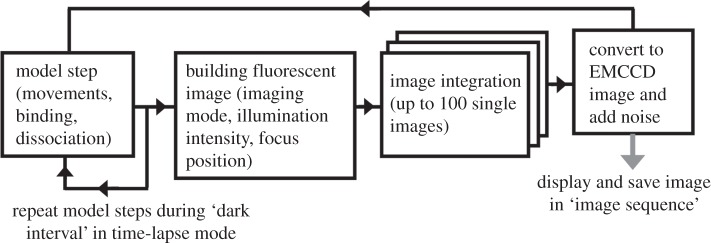
Model flowchart. Basic time step includes calculations of all the movements and possible binding–dissociation events for all the objects present in the system. The *x-, y-, z*-coordinates of all active (light-emitting) objects are used to create a fluorescent image of a cell (if illumination is ‘ON’). The number of images can be summed into a single camera frame to create a realistic image of fast-moving molecules.

During each iteration cycle of the model (figure 1), the physical positions of all the objects are changed according to the motion rules (e.g. random walk, directed movement or static) and the duration of a time step. The possible events, such as binding and dissociation, are executed and the properties of the involved objects are updated. If the ‘cell’ is illuminated at a given time step, then a fluorescent image is built by ‘projecting’ the images of all active (non-bleached) fluorescent molecules to the surface of a virtual imaging device. The size and the brightness of a single fluorophore image will depend on its point spread function (PSF) and on its *x-, y-, z*-position, which determines the amount of incident light the object receives. If required, a number of sequential time steps can be summed to create an averaged fluorescence image which would better represent fast-moving objects. Some random number of normally distributed ‘noise counts’ can be added to every pixel in the image to simulate background fluorescence and camera noise. The ‘raw’ fluorescence image can be converted into a synthetic EMCCD image by multiplying the value of every pixel by a random number. The average of these normally distributed random numbers is determined by a camera ‘gain’.

**Figure 2.**
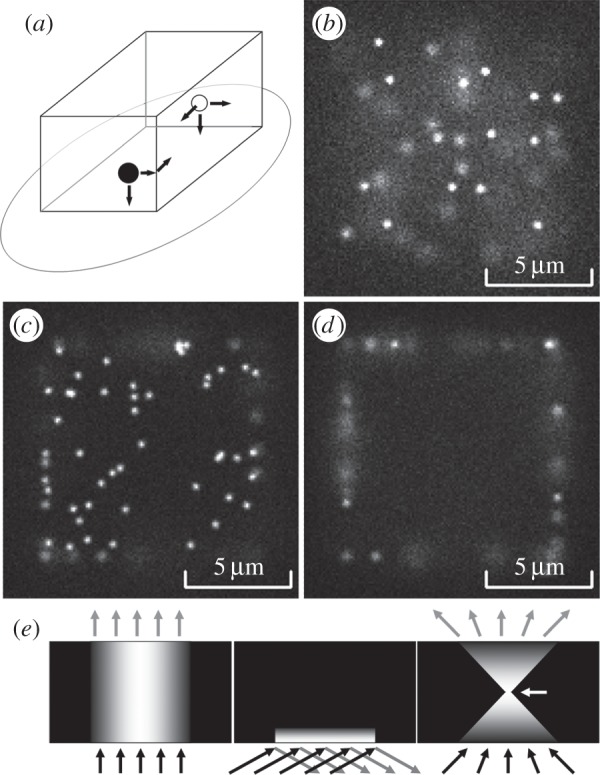
Basic model. (*a*) The ‘cell’ geometry. Molecules of one class (open circles) move freely inside the cell volume but cannot leave it. Molecules of another class (filled circles) move only at the surface of the ‘cell’. An ellipse is the surface of substrata (coverslip) and a default focal plane at *z* = 0. (*b*) Simulated fluorescent image of a cell with molecules randomly moving in the cytoplasm (epi-illumination, focal plane at *z* = 0 µm, cell size 10 × 10 × 10 µm^3^, concentration 2 nM). (*c*) An image of a ‘cell’ with molecules moving at the membrane (epi-illumination, focal plane at *z* = 0 µm, density 1 molecule µm^−2^). (*d*) The same ‘cell’ as shown in (*c*), but the image is built at a focal plane set above the substrate (*z* = 1 µm). Only a few molecules can be seen as sharp spots ‘in focus’, whereas other objects are ‘blurred’ because they are positioned above or below the focal plane. (*e*) Illumination profiles: epi-illumination mode (left); TIRFM (middle); confocal microscopy (right). Arrow points to a focus position.

The synthetic biological ‘cell’ is represented as an enclosed volume in a shape of a parallelepiped with arbitrary *x, y, z* sizes (figure 2*a*). The simulated ‘cell’ is placed on a ‘coverslip’ (an ellipse in figure 2*a*). The interface between the ‘cell’ and the ‘coverslip’ defines the focal plane at *z* = 0.

### 2.1. Object movements

The objects inside the ‘cell’ normally undergo an unrestricted ‘random walk’ type of diffusion limited by the ‘cell’ boundaries. Object movements in all three dimensions (*x, y, z*), are modelled independently [22] using a Gaussian random number generator (GRNG), based on the Box–Muller algorithm (default output *δ* = 1, *μ* = 0) [29]. Mean-squared displacement (MSD) in one dimension is calculated as 2*D*_Δ_*t*, where *D* is the diffusion coefficient, and Δ*t* is a time parallelepiped step [30]. The root-mean-squared displacement (RMSD) was used as a multiplier in the GRNG to generate individual displacement values. The object's *x-, y-, z*-coordinates were stored as floating-point values to avoid bias caused by rounding of integer numbers.

Objects moving on the cell ‘membrane’ (another class) undergo two-dimensional random walk. The same algorithm as above was used to calculate the object displacements, but only in two dimensions. Note that each random displacement value was generated separately for each dimension, so if an object, moving on one side of a ‘cell’, reaches an edge, then it continues its movement on the adjoining side of the parallelepiped (figure 2*a*). Molecules bound to each other have the same physical coordinates (*x, y, z*) and move together each time parallelepiped step, so that the displacement values are calculated once for the pair of objects. The changes in the mobility pattern of the paired objects are explained in §2.

### 2.2. Interactions between the objects

Molecules of the same or different species can be allowed to bind each other. Binding events could happen when the corresponding ‘binding sites’ on both objects are unoccupied and the number of collisions between the two objects reaches a certain threshold. Collisions would occur if two molecules move close to each other, so that the distance between their centres became smaller than the interaction distance (ID) or reaction radii [22]. The simplest way to simulate this process is to reduce the model time step, so that the average amplitude of movements is smaller the ID. Molecules separated by a distance less than or equal to the ID will have at least one collision per time step. If we increase the time step, then the objects will move longer distances, and we will underscore the collision events, because we will not know whether the trajectories of the objects were within the ID or not during the time step.

*A practical example*. A membrane-bound molecule with *D* = 0.1 µm^2^ s^−1^ has RMSD ≈ 6.3 nm at Δ*t* = 100 µs, and ≈2 nm at Δ*t* = 10 µs (MSD = 4*D*_Δ_*t*). Therefore, a 10 µs time step is sufficient to simulate binding processes at ID ≥ 5 nm. However, molecules move much faster within the cytoplasm, where protein *D* is in the range 2–20 µm^2^ s^−1^ [10,11,31], and, for example, with *D* = 5 µm^2^ s^−1^, RMSD ≈ 17.3 nm at Δ*t* = 10 µs, and ≈5.5 nm at Δ*t* = 1 µs (MSD = 6*D*_Δ_*t*; [30]).

Thus, the above-mentioned approach [22] will consume substantial computational time, because we might want to model collisions of hundreds or even thousands of molecules over a reasonable period of time (e.g. 10–100 s). Another approach, proposed by Tournier *et al.* [23], uses probability-based description of the reaction process, suitable for the longer time steps. A simplified version of this approach, requiring a minimal number of calculations, was developed for the present model.

To calculate the probability of binding between any potential pair of objects we need to calculate the average interaction time (AIT)—that is, the time which this couple would spend within the ID during the current time step. For example, we can assume that molecules A and B are point-like objects randomly moving in one-dimensional space; A and B are separated by a distance *x*; we can consider molecule A as a fixed target, whereas molecule B has a mobility of *D* = *D*_A_ + *D*_B_. We can calculate the probability of finding molecule B between *x* and *x* + _d_*x* at a time *t* as [30]
2.1
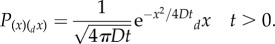



If we assume that *_d_x* = ID we can calculate the probability of finding molecule B within the ID of molecule A at time *t*. We can use discrete integration of the above function over time to find the AIT for any given *x*. Similar equations can be used to calculate the AIT for objects moving in two- and three-dimensional space, but, in these cases, we replace the ID with interaction area (IA = *πr*^2^), and interaction volume (IV = 4/3*πr*^3^), where *r* = ID/2 [23],
2.2
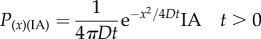

and
2.3
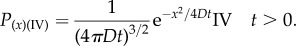



**Figure 3.**
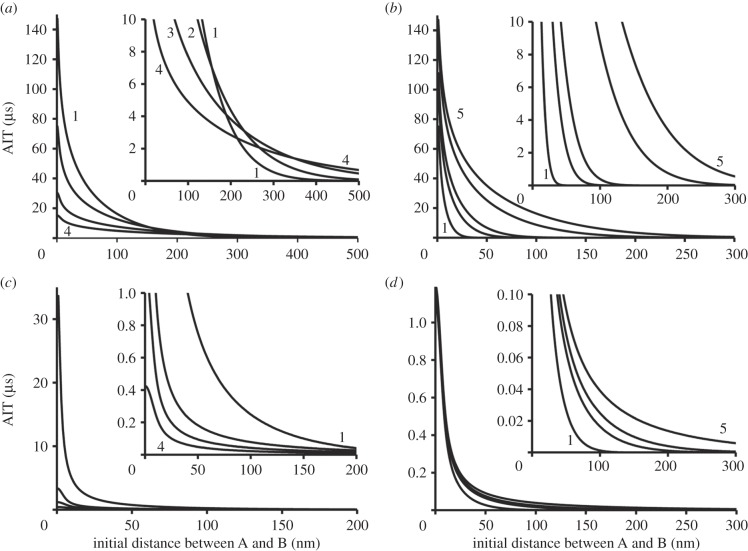
Average interaction time (AIT). (*a*) Objects A and B moving in two-dimensional space have summed mobility *D*: 0.1 (1), 0.2 (2), 0.5 (3) and 1.0 (4) µm^2^ s^−1^ (ID = 5 nm, time step 100 ms). The graphs 1–4 show how AIT depends on the initial distance between A and B (at the beginning of a time step). The inset shows the zoomed part of the same graph. (*b*) Objects A and B moving in two-dimensional space have summed mobility 0.1 µm^2^ s^−1^ (ID = 5 nm). The graphs show how AIT depends on the initial distance and the duration of a time step. The graphs 1–5 (from left to right) were calculated at a time step: 1, 5, 10, 50 and 100 ms, respectively. Inset shows the zoomed part of the same graph. (*c*) Objects A and B moving in three-dimensional space have summed mobility *D*: 0.1 (1), 0.5 (2), 1 (3) and 2 (4) µm^2^ s^−1^ (ID = 5 nm, time step 100 ms). The inset shows the zoomed part of the same graph. (*d*) Objects A and B moving in three-dimensional space have summed mobility 1.0 µm^2^ s^−1^ (ID = 5 nm). The graphs 1–5 (from left to right) calculated at a time step: 1, 5, 10, 50 and 100 ms, respectively. Inset shows the zoomed part of the same graph.

Integrating the probability density function for all possible pairs of objects each time step is unnecessary. Instead, we can build look-up tables containing the AIT values for each class of molecule allowed to bind each other. The index of the look-up table (integer value in ‘nm’ units) can be used to match the distance *x* separating objects at the beginning of a time step and AIT. These tables need to be recalculated only when we change the mobility, or ID, or a time step. The example in figure 3*a* shows how AIT depends on mobility of the membrane-bound objects (two-dimensional diffusion) at a time step of 100 ms (ID = 5 nm). Figure 3*b* shows how the AIT would depend on the duration of a time step if, for example, we simulate dimerization of the membrane-bound molecules. It is clear that the AIT decreases dramatically when the initial distance between molecules increases or when we reduce a time step. In many cases, it will be unnecessary to check the probability of binding for the objects placed at a distance greater than 1 µm apart. Examples in figure 3*c,d* show the AIT for the molecules moving in a three-dimensional space.

Because we know the AIT, we can use a ‘binding rate coefficient’ or ‘reaction rate’ *R*_bind_ (s^−1^) to calculate the probability of binding *P*_bind_ = AIT · *R*_bind_. It was assumed that *R*_bind_ is always smaller than the diffusion-limited binding rate [22,32,33], especially in the case of ligand binding where it depends on the orientation of the small binding pocket at the moment of collision [33], so that more than one collision is required for a successful binding. Therefore, we can use a linear random number generator (with output proportional to *P*_bind_) to calculate the outcome of a binding test for any potential pair of molecules.

Bound molecules dissociate according to a simple zero-order chemical kinetics [32], which is modelled as a random process. The probability of dissociation was calculated as *P*_diss_ = *R*_diss_ · Δ*t*, where *R*_diss_ is the dissociation rate constant. The *R*_bind_ and *R*_diss_ can be changed during a model run to simulate, for instance, some real responses to biological stimulation.

### 2.3. Illumination of a specimen

In order to calculate the number of photons (*N*_ph_) emitted by a fluorescent tag during each time step and calculate the probability of bleaching we need to know the illumination intensity at the *x, y, z* location of an object. There are three major illumination modes (figure 1*e*) which are used in the present model: epi-illumination, TIRFM illumination and confocal illumination.

#### 2.3.1. Laser epi-fluorescent illumination

Laser epi-fluorescent illumination usually uses a TEM_00_ laser beam which has a two-dimensional Gaussian profile (figure 2*e*, left panel) measured by its full width at half maximum (FWHM). It is assumed that the illumination intensity *I*(*x,y,z*) is constant in a *z*-direction, because we are dealing with a transparent specimen. We can use a two-dimensional Gaussian function to calculate intensity *I*(*x,y,z*) in any point of a specimen,
2.4
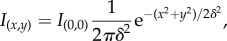

where *δ* is a standard deviation (for Gaussian function FWHM = 2(2ln(2))^½^ · *δ* **≈** 2.4 · *δ*).

#### 2.3.2. TIRFM illumination

TIRFM illumination exploits the effect of total internal reflection of light occurring at the interface between high and low refractive index materials when the angle of incident light *θ* is greater than ‘the critical angle’ *θ_c_* = sin^−1^(*n*_1_/*n*_3_), where *n*_1_ and *n*_3_ are the refractive indices of the low- and high-index materials, respectively [12].

If we assume that a TEM_00_ laser beam is used for TIRFM illumination, then it will have a two-dimensional Gaussian profile in the *x,y*-plane (see epi-illumination mode described above), but the illumination intensity will decrease exponentially in the *z*-direction (figure 2*e*, middle panel) above the interface between the two media. The intensity profile in the *z*-direction will depend on the angle and wavelength of the illuminating beam and the refractive indices of both media [12],
2.5
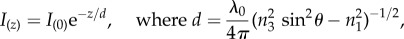

where *λ*_0_ is the wavelength of the incident light in a vacuum, *θ* is the angle of incidental light, and *n*_1_ and *n*_3_ are refractive indexes of the liquid and the solid, respectively. Depth *d* is independent of polarization of an incident beam and decreases with increasing *θ*. Except for *θ* → *θ*_c_ (where *d* → ∞), *d* is of the order of *λ*_0_ or smaller [12]. We can calculate the intensity at any point of a specimen under TIRFM illumination as
2.6
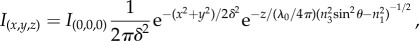

where *I*_(0,0,0)_ is an intensity in the centre of the field at the coverslip level, and *δ* is a standard deviation.

**Figure 4.**
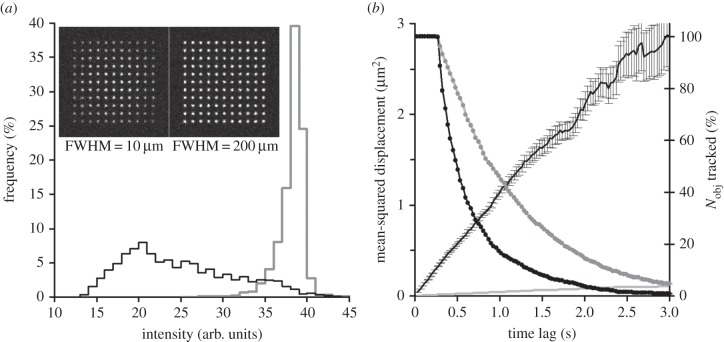
Objects moving at the plasma membrane. (*a*) Distribution of intensities of the tracked objects, illuminating beam FWHM = 10 µm (black), and FWHM = 200 µm (grey). The insets show the first frame in the record before molecules (originally placed in a chessboard pattern) started to move (see the electronic supplementary material, video S4). (*b*) Mobility of the objects moving at the plasma membrane. MSD–Δ*t* (time lag) plot (±s.e.m.) for single molecule objects moving at *D*_lat_ = 0.3 µm^2^ s^−1^ (black line) and 0.01 µm^2^ s^−1^ (grey line). Note that error bars on a grey line are too small to be visible at this scale. Right axis—the fraction of objects tracked at the increasing time lags (Δ*t*) (black dotted line, *D*_lat_ = 0.3 µm^2^ s^−1^; grey dotted line, *D*_lat_ = 0.01 µm^2^ s^−1^). The photobleaching rate was the same in both cases (*R*_bleach_ = 0.5 s^−1^).

The flatness of the *x,y*-plane of illumination in epi-illumination and TIRFM modes depends on the FWHM of a TEM_00_ laser beam. The profile can be virtually flat if the beam FWHM ≫ the *x, y* sizes of the illuminated area, but if the laser beam is narrow (FWHM ≤ the *x, y* sizes of a cell) then the illumination profile will be uneven, which can severely affect the results of measurements (see example in figure 4*a*). Note that if the simulated laser beam differs from the TEM_00_ mode beam, other functions should be used to describe its intensity profile in the *x*, *y* plane. The diameter of the illumination field can be ‘truncated’ by a circular ‘diaphragm’ or by a mask of any other shape or size.

#### 2.3.3. Confocal illumination

Confocal illumination (figure 2*e*, right panel) is the simplest to model because it is a scanning method, where every effort is made to ensure that illumination intensity *I*_(*x,y*)_ is the same at each *x,y-*point illuminated during the scan cycle. It is assumed that a high numerical aperture objective lens (NA ≥ 1.4) is used for imaging, so that the illumination intensity is reduced above and below the focal plane as *I*(*z*) = *I*(*z_f_*) · (*Sz_f_*/(*Sz_f_* + π · (tan(*θ*) · |*z* − *z_f_*|)^2^), where *I*(*z_f_*) is the illumination intensity at focal plane *z_f_*, *Sz_f_* is the size of the focused beam at *z_f_*, and *θ* is half of the angle of the illuminating cone of light (figure 2*e*, right panel) defined by a NA of the simulated objective lens.

### 2.4. Building the fluorescent image

The image of the cell is generated by summing emission from all the fluorescent molecules. In general, the photons emitted by each single fluorophore (point source of light) are spread according to a realistic PSF. If we assume that our system is equipped with an ideal objective lens, then the PSF of a single fluorophore will be approximated by a two-dimensional Gaussian function (equation (2.4)), where *I*_(0,0)_ is the intensity in the centre of the spot. FWHM of a point source of light is roughly equal to half of a wavelength of the simulated colour (FWHM = 0.6*λ*/NA, where *λ* is a wavelength and NA is the numerical aperture of the objective lens). The average number of photons (*N*_ph_) emitted by a single fluorophore is determined by the illumination intensity at its location: *N*_ph(*x,y,z*)_ = *I_xyz_* · *N*_ph(0,0,0)_. A linear random number generator is used to simulate the ‘photon noise’ characteristic for single fluorophore emission, so that the range of *N*_ph_ fluctuations is (*N*_ph_)^1/2^. If an object moves above or below the focal plane of an objective lens, then the size of its image increases. GRNG was used to generate *x-* and *y*-coordinates of each of the *N*_ph_ photons accumulated in the corresponding ‘pixels’ of a virtual CCD camera. The FWHM value used as the GRNG multiplier was determined by the distance between the *z*-coordinate of an object and a focal plane. In the case of an ideal objective lens, the FWHM value would increase according to a simple hyperbolic function: FWHM(*z*) = *γ*_obj_ · (*z* − *z_f_*)^2^ + FWHM*_f_*, where *γ*_obj_ is a coefficient defined by the magnification of an objective lens and its numerical aperture, *z_f_* is the focal plane, and FWHM*_f_* is the size of a fluorophore image at *z* = *z_f_*. *γ*_obj_ is equal to ∼0.6 µm^−1^ for the objective lens × 100 NA 1.45 (see §1 in the electronic supplementary material).

In the case of confocal imaging, a significant fraction of photons, emitted by fluorescent molecules placed above or below the focal plane, does not reach the detector because they are blocked by a pinhole. Therefore, these molecules also generate a smaller number of photons than the molecules placed at the focal plane: *N*_ph_(*z*) = *N*_ph_(*z_f_*)/(1 + *γ*_PH_ · (*z* − *z_f_*)^2^), where *N*_ph_(*z_f_*) is an average number of photons detected at the focal plane *z_f_*, and *γ*_PH_ is a coefficient determined by the size of the pinhole and the magnification of the objective lens.

In real experiments, some fluorescent molecules will be bleached during the observation period. The photobleaching process is modelled as a simple zero-order chemical reaction, where the probability of bleaching *P*_bleach_ = *R*_bleach_ · Δ*t* · *I_xyz_*, where *R*_bleach_ is the photobleaching coefficient (s^−1^) and Δ*t* is the model time step. *R*_bleach_ can be set before and changed during the model run. Note that if a single molecule object has few fluorescent tags, then each of them would undergo the bleaching process independently of others.

### 2.5. Single particle tracking

The sequences of fluorescent images were analysed by the single particle-tracking software GMimPro [19]. The results of automatic detection and tracking were presented as individual trajectories yielding information about mobility and intensity of each individual object. Some single molecule objects were not detected because their trajectories were shorter than the minimal trajectory length (20 data points) set during the detection phase of the analysis. This would happen because the images of individual moving molecules overlapped, or because molecules were bleached, or because molecules moved to the unilluminated parts of the cell (e.g. above the basal side of the cell illuminated in TIRFM mode).

### 2.6. Default conditions

In the examples presented below, the model was run with the cell sizes = 10 × 10 × 10 µm^3^, imaging rate 33 fps (10 model time steps summed per frame). An objective lens × 100/1.45 NA was simulated giving a final magnification of 100 nm pixel^−1^. Single fluorophore image size (FWHM) was set to 250 nm, emission rate 6000 photons s^−1^ in the centre of the illumination area at *z* = 0, where the photobleaching rate *R*_bleach_ = 0.5 s^−1^. EMCCD gain = 5 (average number of counts approx. 30 000 fluorophore^−1^ s^−1^), noise level 2 counts pixel^−1^ (RMS). The illuminating beam FWHM = 100 µm, TIRFM illumination incident angle 64°. The mobility of molecules moving in the cytoplasm was set to 5 µm^2^ s^−1^ (concentration 2 nM), and the mobility of the membrane-embedded molecules was 0.3 µm^2^ s^−1^ (density 1 molecule µm^−2^). The list of examples showing some specific conditions is presented in table 1. All these parameters can be changed before the model run and some during the run (see electronic supplementary material).

**Table 1.** A list of video examples generated by the model.

scenario	illumination	specific conditions	video	references
free moving intracellular molecules	epi. TIRFM	concentration 2 nM, *K*_diff_ 5 µm^2^ s^−1^	S1 S2	[10,11]
molecules moving at cell membrane	epi. TIRFM	density 1 µm^−2^, mobility 0.3 µm^2^ s^−1^	S3 S4	[13,19,24,28]
barriers at cell membrane	TIRFM	grid sizes 1 × 1 µm^2^	S5	[2]
lipid rafts on cell membrane	TIRFM	raft sizes 1 × 1 µm^2^, distance between rafts 1 µm	S6	[7]
intracellular molecules (A) binding to molecules at cell membrane (B)	gated TIRFM, 10 fps	mobility of (A) 5 µm^2^ s^−1^, mobility of (B) 0.001 µm^2^ s^−1^, *R*_bind_ 1 × 10^6^ s^−1^, *R*_diss_ 0.05 s^−1^	S7	[10,13,14,31]
free moving membrane molecules (A) binding to immobile anchors (B)	TIRFM	density of (A) 0.5 µm^−2^, density of (B) 0.5 µm^−2^, *R*_bind_ 1 × 10^5^ s^−1^, *R*_diss_ 1 s^−1^	S8	[3,25,27,34]
transient dimerization of membrane-bound molecules^a^	two-colour TIRFM	‘green’ 1 µm^−2^, ‘red’ 1 µm^−2^, mobility 0.1 µm^2^ s^−1^, *R*_bind_ 1 × 10^5^ s^−1^ , *R*_diss_ 1 s^−1^	S9	[6,24,35]
photobleaching of tetramers at cell membrane^a^	TIRFM	density 1 tetramer µm^−2^, mobility 0.001 µm^2^ s^−1^	S10	[8,36]
molecules moving inside tubular network^a^	TIRFM	concentration inside the tubules 5 µM	S11	[37]
molecules transiently binding to microtubules^a^	epi., 5 fps confocal scan	5000 binding sites placed at 8 nm steps, *R*_bind_ 1 × 10^5^ s^−1^ , *R*_diss_ 2 s^−1^/*R*_diss_ 0.02 s^−1^	S12 S13	[21]

^a^These examples are described in the electronic supplementary material.

## 3. Results and discussion

### 3.1. Random diffusion in cytoplasm and at the plasma membrane

In the simplest case, the model would contain only one class of objects. For example, a single frame from the electronic supplementary material, video S1 (figure 2*b*) shows randomly moving non-interacting fluorescent molecules (e.g. green fluorescent protein) inside the cell (epi-illumination mode, concentration 2 nM). Electronic supplementary material, video S2 shows the simulation made under the same conditions, but in TIRFM illumination mode. The focus plane was set at *z* = 0 µm in the first half and then moved to *z* = 0.2 µm in the second half of the record. Another basic case scenario is molecules randomly moving on the cell membrane without interactions with other molecules or obstacles. Electronic supplementary material, video S3 shows the results of a simulation carried out in epi-illumination mode at a density of 1 molecule µm^−2^. In the first half of the record, the focal plane was set to *z* = 0 µm (figure 2*c*) and it was then increased to *z* = 1 µm in the second half of the record (figure 2*d*). Unlike the previous case, individual membrane-bound molecules were present on the image sequence for some significant time. Electronic supplementary material, video S4 was made under the same conditions, but in TIRFM mode.

The imaging conditions can affect both the appearance of fluorescent molecules (brightness and shape) and the results of their detection. Figure 4*a* shows how the shape of the illuminating beam can affect the results of detection and tracking. When a narrow laser beam was used to illuminate membrane-bound molecules (figure 4*a*, black line and left inset), the distribution of intensities of tracked molecules was much wider than with the nearly-flat illumination conditions (FWHM = 200 µm). In the last case, the results of automatic tracking had a narrow distribution of intensities of detected and tracked objects (figure 4*a*, grey line). This conclusion is correct for both TIRFM and epi-illumination microscopy modes. The averaged mobility of automatically tracked objects was 0.298 µm^2^ s^−1^ and the averaged MSD–Δ*t* plot had a linear shape (figure 4*b*, black line) characteristic of freely diffusing molecules. The grey line in figure 4*b* shows the averaged MSD–Δ*t* plot for a record made under the same conditions, but the mobility was reduced to 0.01 µm^2^ s^−1^. The two exponentially decaying lines (right *y*-axis) in figure 4*b* show the number of objects which contributed data to the MSD–Δ*t* plot at the increasing time intervals. The fitted off-rate or the rate of losing tracked objects was much higher for the fast-moving objects (1.25 s^−1^ at *D*_lat_ = 0.3 µm^2^ s^−1^) than for the slow-moving objects (0.55 s^−1^ at *D*_lat_ = 0.01 µm^2^ s^−1^), where the off-rate was close to the photobleaching rate (*R*_bleach_ = 0.5 s^−1^). This example shows that even under simple conditions the results of analysis (e.g. calculated bleaching or dissociation rate) can be severely affected by the imaging conditions—overlapping of the images of fast-moving molecules leads to erroneous truncation of the trajectories of detected objects. Choosing cells with lower densities of molecules or decreasing the temperature [28] would allow us to measure the off-rate free from the artefacts of overlapping moving objects.

### 3.2. Anomalous diffusion at the plasma membrane

**Figure 5.**
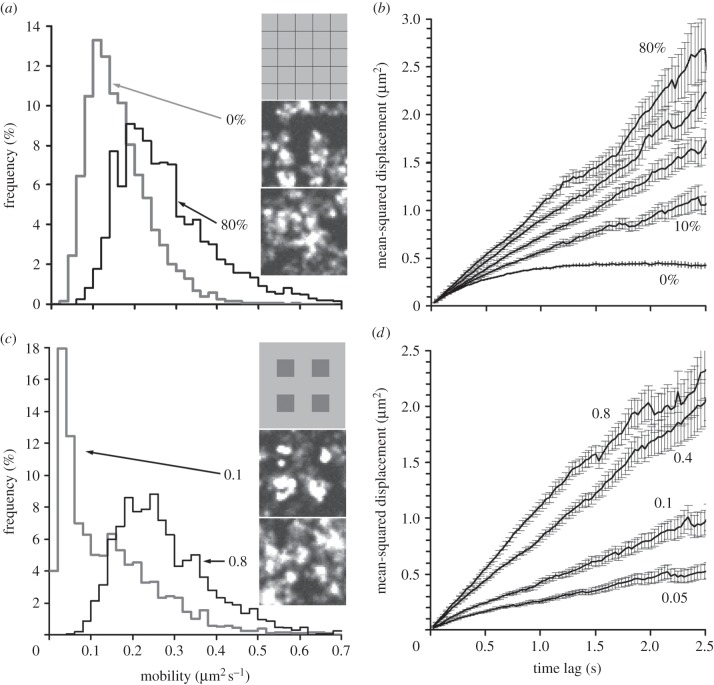
Anomalous diffusion. (*a*) Distribution of motilities of individual randomly moving objects (*D*_lat_ = 0.3 µm^2^ s^−1^) at a membrane with partially permeable ‘barriers’ (1 × 1 µm^2^ grid size). Grey line, permeability 0% (measured *D*_lat_ = 0.163 µm^2^ s^−1^); black line, permeability 80% (measured *D*_lat_ = 0.276 µm^2^ s^−1^). Insets show the relative grid size (top) and the corresponding (averaged over 100 frames) images at permeability of 0% (middle) and 80% (bottom). (*b*) MSD–Δ*t* (time lag) plots (±s.e.m.) at increasing levels of permeability (0%, 10%, 20%, 40% and 80%). (*c*) Distribution of motilities of individual randomly moving objects (*D*_lat_ = 0.3 µm^2^ s^−1^) at a membrane containing ‘lipid rafts’ (size 1 × 1 µm^2^). Grey line, mobility inside the ‘lipid rafts’ was set to be 0.1 of normal mobility (measured *D*_lat_ = 0.141 µm^2^ s^−1^); black line, mobility inside the ‘lipid rafts’ was set to 0.8 of normal (measured *D*_lat_ = 0.278 µm^2^ s^−1^). Insets show the relative raft size (top) and averaged over 100 frames images at a ratio of mobility in the rafts of 0.1 (middle) and 0.8 (bottom). (*d*) MSD–Δ*t* plots (±s.e.m.) at different ratios of mobility inside the ‘lipid rafts’ (the ratio values shown next to the graphs).

The movement of molecules associated with the plasma membrane can be affected for a number of reasons. One type of potential restriction is the presence of ‘barriers’ or ‘fences’ creating compartments at the plasma membrane [2,16,17,38]. In this case, the slope of the MSD–Δ*t* plot will decrease at higher Δ*t* and become horizontal at MSD values close to 40% of the size of a compartment (see equations 11–13 in [2]). This scenario was simulated by dividing the cell membrane into square compartments (figure 5*a*, top inset), using virtual ‘barriers’ restricting the objects' movements into any neighbouring compartment. The example in figure 5*a*,*b* shows that if the barriers are completely impenetrable, the average mobility decreases twofold (figure 5*a*, grey line, and the electronic supplementary material, video S5) compared with the freely diffusing molecules described previously (figure 4*b*). The MSD–Δ*t* plot became horizontal at MSD values close to 40% of the size of the compartment. However, if the barriers became partially permeable, the MSD–Δ*t* plot became more linear (figure 5*b*), which makes it difficult to distinguish between the slow diffusion and fast diffusion limited by the barriers.

Another potential factor limiting movement at the cell membrane is the presence of lipid rafts—that is, patches of membrane that have high viscosity compared with the surrounding membrane [7]. The simplest case of a lipid raft scenario is shown in figure 5*c*, top inset, where square patches are placed at equal distances from each other. On the example shown in figure 5*c*,*d,* 1 × 1 µm^2^ patches were separated by 1 µm gaps, so the rafts cover 25% of the cell membrane. The single molecule objects randomly seeded on the membrane at the beginning of the run were concentrated in the raft areas during the run. The rate and degree of crowding would depend on the ratio of the mobility inside/outside the raft patches (*R*_raft_) and the absolute mobility value. If the ratio was large (*R*_raft_ = 0.1), then we would observe rapid accumulation of molecules in raft patches (figure 5*c*, middle inset, and the electronic supplementary material, video S6). After an initial period of equalizing, the distribution of the mobilities of tracked molecules became bimodal (figure 5*c*, grey line), reflecting the fact that the majority of molecules were concentrated inside the rafts [7]. It is important to note that many molecules inside the crowded patches were not detected and therefore were excluded from the data analysis. The effect of the presence of the rafts was less detectable when the mobility inside the rafts was close to the mobility of the surrounding membrane (*R*_raft_ = 0.8; figure 5*c*, black line). The shape of the averaged MSD–Δ*t* plot remained linear (figure 5*d*) in a wide range of mobility ratios (0.05–0.8). Thus, analysis of the distributions of mobility and detection of clusters of molecules on the membrane is the best method for detecting potential lipid rafts or other types of membrane patches with increased viscosity.

### 3.3. Binding kinetics

**Figure 6.**
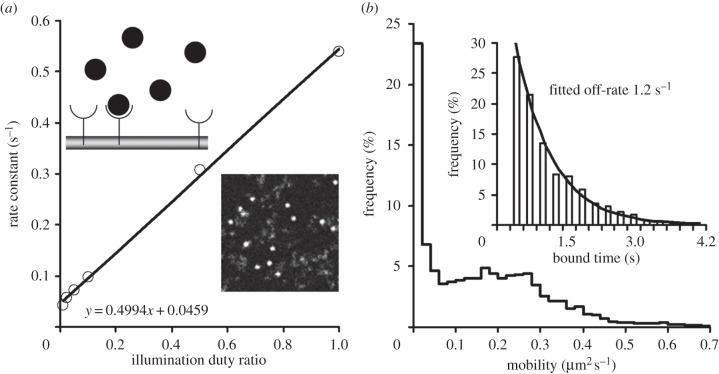
Interaction between molecules. (*a*) Binding of free moving intracellular molecules to molecules at the cell membrane. The dissociation and photobleaching rates were measured by systematically changing the illumination duty ratio and measuring the off-rate of molecule disappearance. The slope of the linear regression is the measure of the photobleaching rate and the offset value is the measure of the dissociation rate. Top inset shows a schematic representation of non-fluorescent molecules (semicircle) at the membrane and fluorescent molecules (filled circles) moving in the cytoplasm above it. Bottom inset shows an image (10 × 10 µm^2^) from a record where some fluorescent molecules were bound to the sites at the membrane (bright round spots) while some others were moving freely in the cytoplasm (dim cloud-like objects). (*b*) Distribution of the mobilities of 4113 objects moving at the cell membrane and binding to immobile anchors (*R*_bind_ = 1 × 10^5^, *R*_diss_ 1 s^−1^). Inset shows the distribution of ‘bound’ times for 358 objects which showed clear temporal immobilization during the model run (black line is a single exponential fit to this distribution).

Many classes of intracellular molecules can bind to the plasma membrane and become membrane bound for some period of time [10,14,15,31] (figure 6*a*, top inset). The following example simulates transient binding of the Pleckstrin homology (PH) domain of molecular motor myosin-10 to specific phosphoinositol phospholipids at the plasma membrane [14]. Because PH molecules bound to a myoblast's membrane have very limited mobility, it was possible to use the gated-illumination time-lapse imaging mode to measure the dissociation rate. The binding rate (*R*_bind_) was set to 1 × 10^6^ s^−1^ and *R*_diss_ = 0.05 s^−1^. The concentration of fluorescent PH-domain molecules was 2 nM, and the density of non-fluorescent ‘phosphoinositol’ targets was 1 µm^−2^. Under these conditions, about 15% of PH-domain molecules were bound to the plasma membrane (figure 6*a* and the electronic supplementary material, video S7). To measure the dissociation and photobleaching rates, the model was run in the time-lapse mode (figure 1) with increasing dark intervals between recorded images. The off-rate (the rate of disappearance of the fluorescent spots ‘landed’ on the membrane during recording) was measured and plotted against the illumination duty ratio. The slope of the fitted regression line was a measure of the photobleaching rate (0.499 s^−1^), and the intercept of the regression line with the *y*-axis was a measure of the dissociation rate (0.046 s^−1^) [14].

Some molecules, moving freely on the plasma membrane, can bind to other molecules of the same (e.g. form dimers [6,24,35]) or other (e.g. bind to immobile anchors [3,25,27,34]) species. The last case scenario can be modelled by the introduction of the two classes of membrane-bound molecules (one mobile and one immobile) and by allowing molecules of one class to bind molecules of another class. For example, randomly moving membrane-bound fluorescent molecules (density 0.5 µm^−2^, *D*_lat_ = 0.3 um^2^ s^−1^) bind to immobile non-fluorescent molecules (density 0.5 µm^−2^) randomly placed at the cell membrane (electronic supplementary material, video S8). The proportion of bound/free moving molecules will depend on the binding and dissociation rates. At *R*_bind_ = 1 × 10^5^ s^−1^ and *R*_diss_ = 1 s^−1^, 45% of mobile molecules were bound to immobile counterparts (approx. 10% at *R*_bind_ = 1 × 10^4^ s^−1^). The results of detection and tracking, at *R*_bind_ = 1 × 10^5^ s^−1^, showed a bimodal distribution of the mobilities of the detected objects (figure 6*b*), where 23% of objects were completely immobile (average *D*_lat_ = 0.16 um^2^ s^−1^). The fraction of tracked objects which showed a detectable period of immobilization (mobile–immobile–mobile pattern) was small (358 of 4113, or approx. 9% of all objects detected in 10 model runs). The distribution of ‘bound’ time had an exponential shape (fitted off-rate 1.2 s^−1^; figure 6*b*, inset). Because after the moment of dissociation the tracked molecules were still visible, we do not have to take into account the effect of photobleaching.

### 3.4. Discussion

This model uses the advantages of object-oriented programming [29,39] to model diverse populations of single molecules represented as objects of a given class. This approach makes it easy to modify and extend the basic model in order to simulate new types of experiment (for example, creating new ‘child’ classes of single molecules moving on filaments from the ‘parent’ class of cytoplasm-based molecules) or to use specific parts of the model (e.g. one specific class and one type of imaging condition).

The continuous space model, which uses floating-point physical coordinates, was chosen over the matrix-type model [34,37] because it was not limited by the size of the matrix elements in a three-dimensional space and, therefore, allowed modelling of very small changes in the object's position (less than 1 nm). The limitation of the matrix-type model is potentially an important issue when tracking objects with subpixel accuracy [19] or modelling long-range movements arising from frequent but small changes in an object's position [21]. The possible introduction of a three-dimensional matrix into the continuous space model would allow representation of complex cell structures (e.g. curved cell membranes, filopodia and intracellular organelles of irregular shape).

Mammalian cells may contain many thousands of fluorescent molecules of one or more species [24,28]. Modelling movements, interactions and fluorescence output for a few thousand objects over a reasonable time period would require substantial computer power. It would be difficult to run such a model on a personal computer 10 years ago. However, nowadays, it possible to complete 1000–2000 image sequences in just a few minutes. For example, a computer equipped with a 3 GHz, i7 processor (Intel Co, CA, USA) runs the present model simulating 1000 cytoplasm-based molecules interacting with 1000 membrane-bound molecules and building a fluorescent image every time step at a rate of approximately 10 cycles per second. So, even at a rate of 100 fps, the model run will be only 10 times slower than real time.

The speed of the model run was increased dramatically by the algorithm calculating the probability of binding between each potential pair of reacting molecules. The solution was to calculate the AIT for the classes of molecules with known mobility and physical sizes. In most cases, the AIT rapidly decreases to negligible values at intermolecular distances greater than 0.5 µm (see figure 3 for details). Therefore, AIT values can be calculated before the model run and stored in a look-up table. This approach greatly reduces the number of calculations during the model run. There was no need to calculate which of the potential pairs was at the shortest distance—the eligible molecules would eventually bind the closest neighbour owing to the sharp increase in the AIT. This algorithm was tested by modelling PH-domain binding to the specific phospholipids on the cell membrane [14], modelling the binding of free moving membrane-bound molecules to the immobile anchors [25,27], and modelling transient dimerization of membrane-embedded molecules [24]. In the case of PH-domain binding to membrane phospholipids, the dissociation rate set in the model (0.05 s^−1^) was found to be very close to the actual measured rate of 0.046 s^−1^ (figure 6*a*, and see figure 4 in [14] in order to compare it with 0.05 s^−1^ from the real data analysis). In the case of membrane-embedded molecules forming transient dimers (e.g. G-protein coupled receptors), the exponential distribution of dimer lifetimes produced an estimated dissociation rate, 1.2 s^−1^, which was slightly higher than the rate of 1 s^−1^ set in the model and was very close to the measured dissociation rate (1.3 s^−1^) of muscarinic M_1_ receptors studied in real two-colour imaging experiments (see figure 4 from [24] and compare it with electronic supplementary material, figure S2). These comparisons allow us to conclude that the proposed model quantitatively reproduces the results of real single molecule experiments.

The examples presented in the Results and Discussion sections demonstrate the importance of realistic illumination and fluorescence output modelling for cross-checking single molecule experiments. It was shown that uneven illumination severely affects the conclusions of single molecule data analysis—figure 4*a* shows that the use of a narrow laser beam for illumination leads to the pseudo-bimodal distribution of intensities of the detected molecules and to the wrong conclusion about the presence of a mixture of monomers and dimers, whereas, in fact, the model sample contained only monomers. The summing of time steps (figure 1) is required to build realistic fluorescent images of fast-moving molecules acquired at a frame rate of 20–100 fps. Otherwise, fast-moving fluorescent molecules would look like static objects, which would affect the data analysis. This procedure is essential when different species of fluorescent molecules are tested in one experiment or when fast-moving intracellular molecules bind slow-moving or static molecules (for example, binding to cell membrane (figure 6*a*) or to microtubules (electronic supplementary material, figure S4*f*)).

This model can also be used for quantitative testing of some specific hypotheses about single molecule dynamics in cells. For example, even a modest level of permeability (10%) of a putative potential membrane barrier [2] would dramatically change the shape of the MSD–Δ*t* plot (figure 5*b*) which is normally used to identify the confined diffusion [38]. Therefore, other methods should be used to probe the presence of barriers on a cell membrane. It has also been shown that single molecules moving at the membrane containing lipid rafts would have a linear shape of an averaged MSD–Δ*t* plot (figure 5*d*), but the shape of the distribution of the mobility of the individual molecules would become bimodal (figure 5*c*, grey line) and the majority of the molecules would concentrate inside the rafts [7] if the level of mobility inside the rafts falls below 10% of the mobility outside the rafts (figure 5*c*, middle inset, and electronic supplementary material, video S6).

The results of modelling of some other important single molecule experiments are presented in the electronic supplementary material. These include the use of the two-colour imaging [24] for testing the monomer–dimer transitions; subunit counting in membrane-bound tetramers [36]; and modelling single molecule dynamics in the presence of intracellular structures.

## Supplementary Material

Supplemental Material

## Data accessibility

Readers can download the full version of this model from www.nimr.mrc.ac.uk/gmimpro to set and test their own experimental scenarios in fully adjusted imaging conditions.
