# Supplementary material for: Single molecule dynamics in a virtual cell: a three-dimensional model that produces simulated fluorescence video-imaging data
Source: J R Soc Interface. 2014 Sep 6;11(98):20140442. doi: 10.1098/rsif.2014.0442 (PMC4233692; doi:10.1098/rsif.2014.0442)
Supplement: Supplemental Material [file rsif20140442supp1.pdf]

Supplemental Material for
"Single Molecule Dynamics in a Virtual Cell: A Three-Dimensional Model that Produces Simulated Fluorescence Video-Imaging Data"

Gregory I Mashanov
Division of Physical Biochemistry, MRC National Institute for Medical Research, Mill Hill, London, NW7 1AA, UK (gmashan@nimr.mrc.ac.uk)

1. Measuring single fluorophore FWHM
2. Example of two-colour imaging
3. Subunit counting example
4. Intracellular structures
5. Simulating cell autofluorescence
6. Model interface
7. Additional references

1. Measuring the size of single fluorophore FWHM relative to its distance from the focal plane.

Imaging: A drop of Phosphate Buffer Solution (PBS) (pH7.4), containing 1 nM of Cy3B (Amersham Pharmacia Biotech, UK), was placed on the cleaned No1 glass coverslip fixed inside rigid imaging chamber [1] for 5 minutes. Unbound fluorescent molecules were washed away few times with PBS which was left in the imaging chamber during observation period. The chamber was placed in the custom-made x-y-z nano-positioner (SLC-17, SmarAct, Germany) above the objective lens (Alfa-plan ×100, NA1.45, Carl Zeiss, Germany). An inverted microscope (Axiovert-S100TV, Zeiss, Germany) was equipped with additional magnifier (Optovar ×1.6, Zeiss, Germany), and custom made TIRFM illumination system [2] (laser 561 nm, 100 mW). Sequences of images were recorded using EMCCD camera (iXon897BV, Andor Technology, UK). During the recording (scale 100 nm pixel^{-1}, rate 20 fps) the imaging chamber was moved up and down (±2 μm) making 100 nm steps made every 10th frame (2 steps s^{-1}).

Measurement: The single fluorescent spots, representing individual Cy3B molecules, were detected on frames at the focus position closest to the coverslip surface using ASPT algorithm implemented in GMimPro tracking software www.nimr.mrc.ac.uk/gmimpro [3]. *x* and *y* coordinates of the centres of the detected spots were used to create an averaged image of all the spots identified in the given record at all given *z*-positions. The averaged images were fitted with the matching 2-D Gaussian mask using least squares method (the FWHM of the mask was changed using 10 nm increment; the amplitude increment was set to 5% of the spot intensity at z-coordinate closest to 0).

The obtained relation between z-coordinate and spot FWHM (Fig. S1) was fitted with simple hyperbolic function: $FWHM(z)= \gamma_{obj} \bullet z^2 + FWHM_0$, where $FWHM_0$ is the Full Width at Half Maximum at the *z* closest to the coverslip surface, and γ_{obj} is the coefficient determined by the magnification and numerical aperture of an objective lens. **Note:** that the best fitted $FWHM_0$ value (0.45 μm) was bigger ~0.3 μm, expected for the tested Cy3B fluorophore (Emission maximum 570nm). This can be explained by the errors associated with the averaging process and by the drift associated with the repetitive vertical movements of the specimen.

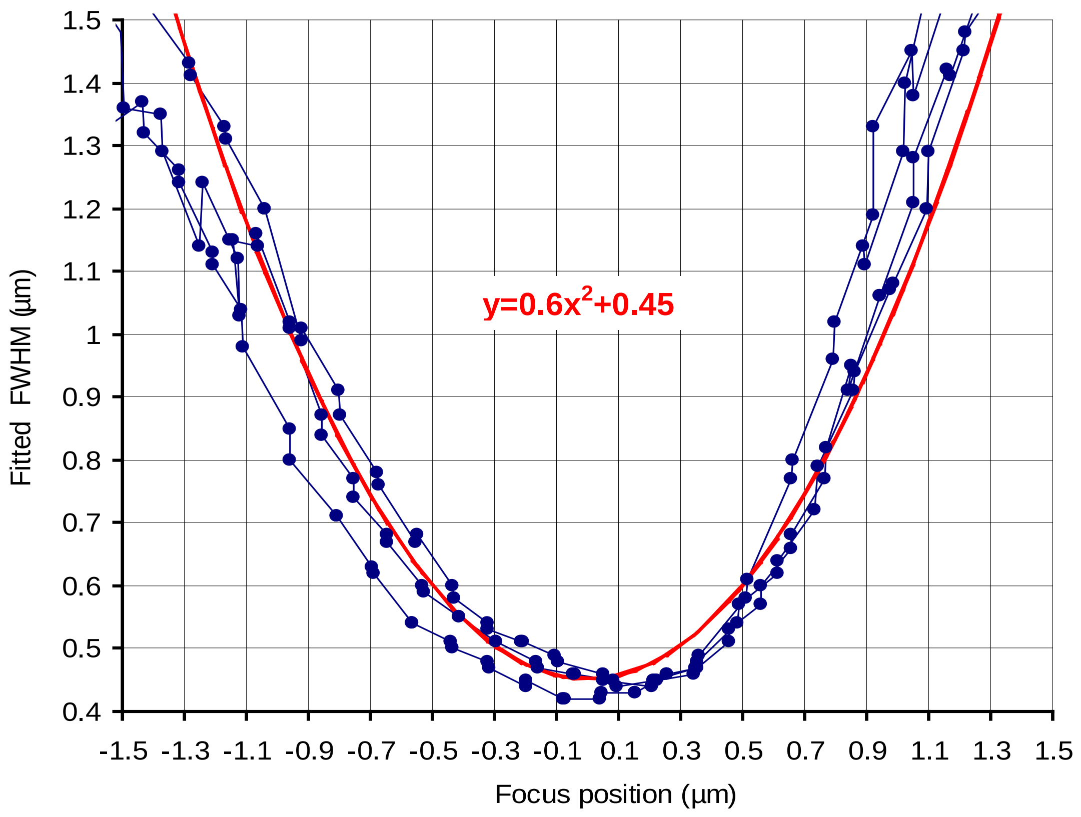

Figure S1. How the size of single fluorophore FWHM depends on its distance from the focal plane. *Single Cy3B molecules were placed on the coverslip surface and imaged while the objective lens was moved up and down by 100 nm each step. Averaged single fluorophore FWHM at each z-position was used to determine the type of the dependence and to measure its coefficient.*

2. Two-colour imaging

Single molecules of the same or different species could have fluorescent tags of different colour (e.g., GFP and RFP) and therefore could be imaged separately, either simultaneously (using image splitter or two cameras) or by using alternating illumination method reported by the Hern and others [4] (fluorophores of one colour are illuminated during one image acquisition and another colour is used during the next image acquisition and so on). Following example simulates muscarinic GPCR family receptors moving on the cell membrane (mobility 0.1 $\mu m^2\ s^{-1}$, density 2 receptors μm^{-2}). Receptors were labelled with either green or red tag (1:1 ratio) and allowed to bind each other ($R_{bind} = 1\times10^5\ s^{-1}$, $R_{diss} = 1\ s^{-1}$). After some period of equilibration, ~35% of molecules were found to be dimers and estimated dissociation rate was 1.2 s^{-1} (See Fig. S2 and Hern and others [4] for the details of the analysis procedure).

Video S9 shows overlapped images of “green” and “red” molecules moving and transiently binding to each other. Note: that this record was modelled in the alternating illumination mode so that the acquisition of green and following it red image is separated by 30 ms (16.5 fps per channel), therefore some green and red spots representing a two-colour dimers do not overlap completely. The Fig.S2A is a two-colour image taken at the beginning of the record. It shows some number of yellow spots which represent “green”-“red” dimers. Fig. S2B shows 3 representative *x-y* trajectories for the pairs of “green”-“red” molecules transiently binding each other during observation period. There is also similar number of bright green and red spots which represent dimers labelled with two tags of the same colour.

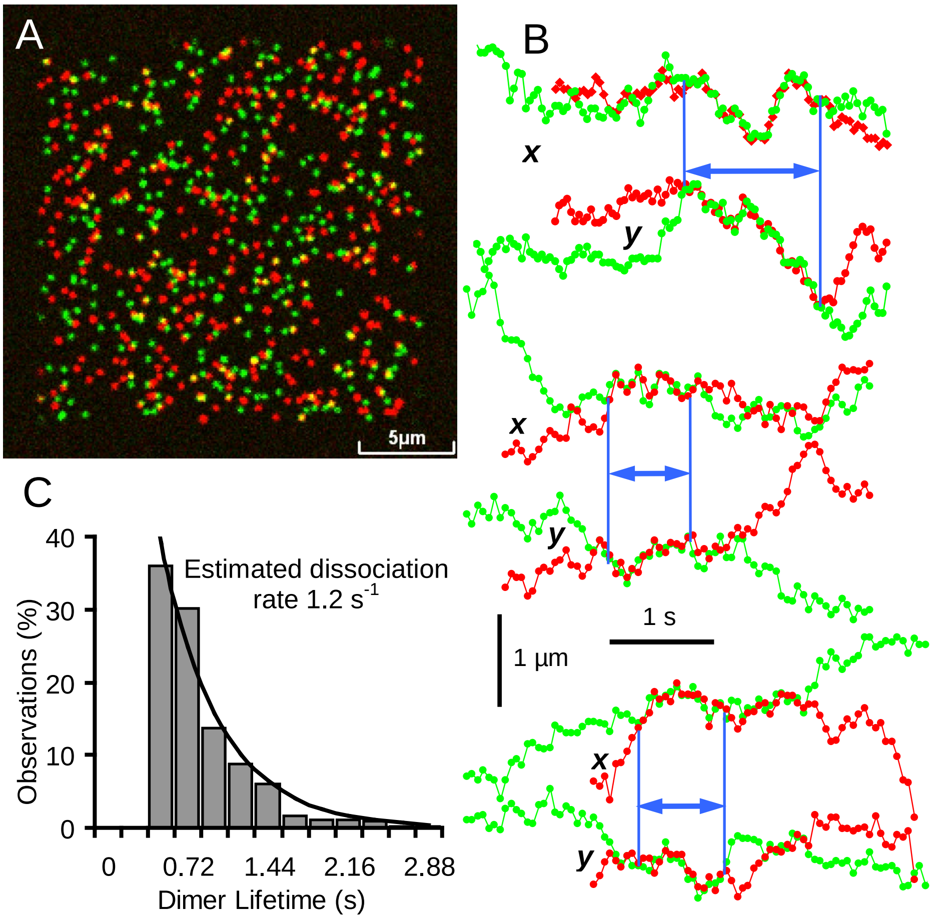

Figure S2. Dimerisation of the membrane receptors modelled in two-colour imaging mode. A. *An overlap of the first “green” and “red” images in the record.* ***B.*** *3 pairs of representative x-y single molecule trajectories show brief periods of dimerisation (blue arrows) when two molecules move together.* ***C.*** *A histogram of the lifetime of 468 dimers collected in 0.24s bins. The solid line is a monoexponential fit for a mean lifetime of 0.5 s.*

3. Subunit counting example.

In some cases single molecule species can be tagged with more than one fluorophore (i.e. constitutive dimers, tetramers, or monomers labelled with few fluorescent tags). Individual bleaching events can be used for counting the number of fluorescent tags / monomers associated with one single-molecule object [5]. In the following example individual molecules, each tagged with 4 fluorophores, were randomly placed on cell membrane (density 1 molecule μm^{-2}). Molecules were set to have very limited mobility (D_{lat}=0.001 μm^{-2} s^{-1}), so that the movements would not affect the detection of photobleaching events (photobleaching rate R_{bleach}=1 s^{-1}, imaging rate 33 fps). Single Fluorophore Detection Algorithm (SFDA) [3], implemented in GMimPro image processor, www.nimr.mrc.ac.uk/gmimpro was used to detect individual photobleaching events and collect intensity histograms in the identified locations (0.5 × 0.5 μm^2) where more than one bleaching event was detected. Fig. S3 shows fluorescence time-course for 10 representative spots identified in one record (Video S10). It is important to note that only fraction of spots (tracks 1, and 3) showed 4 bleaching steps. Other showed smaller number of steps (tracks 2, 7, 8, 9) or more complex fluorescence change caused by the overlapping images of closely placed molecules and small movements of the molecules.

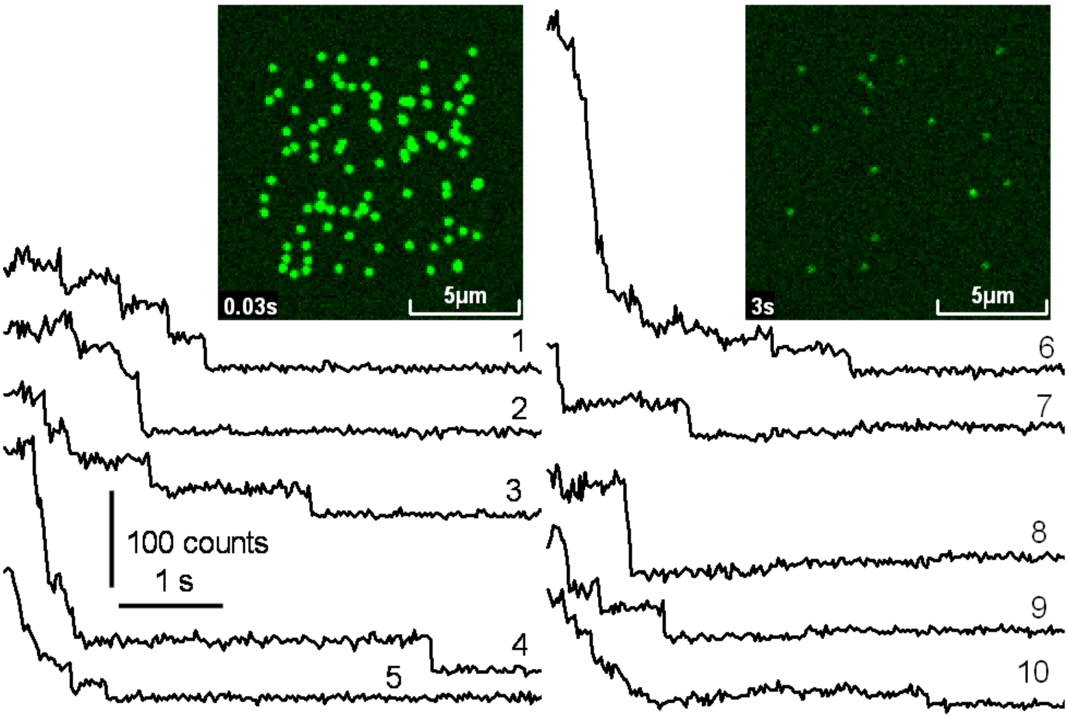

***Figure S3. Counting subunits in the membrane bound tetramers.** Each fluorescent object (molecule) placed on cell membrane had 4 active fluorophores at the beginning of recording / illumination (photobleaching rate set to 1 s^{-1}, imaging rate 33 fps, mobility 0.001 um^2 s^{-1}). Ten representative trajectories show fluorescence time-course for some spots identified in the record. Inserts show fluorescent images of the cell (TIRFM mode) at the beginning, and 3 seconds after beginning of the record.*

4. Intracellular structures.

Some basic intracellular structures, such as nucleus or tubular Golgi network, can be introduced to the basic model without complicated description or significant increase in the number of calculations if such structures can be defined as simple geometrical shapes with the borders impenetrable for molecules moving in the cytoplasm or inside the defined structure.

Fig. S4A shows an epithelial cell containing dark oval nucleus free from fluorescent molecules moving in the cytoplasm, so, in the model nucleus can be represented as an ellipsoid with arbitrary radii r1, r2, r3 [6] (Fig. S4B, top insert). In the following example the concentration of free moving molecules in cytoplasm was 5 nM and the imaging rate was 1000 fps. The averaged epi-fluorescent image (300 frames) was build at the focal plane set at the bottom of the cell (z=0 µm, Fig. S4B, left insert) and at the half cell height (z=4 µm, Fig. S4B, right insert).

Some tubular cellular structures, like endothelial reticulum (See example on the Fig. S4C), can be modelled as interconnected network of round tubes (Fig. S4D, left panel) [7] impenetrable for molecules moving inside the tubes. Example of the Fig. S4D, right panel shows fluorescent molecules inside the network of round tubes (Ø0.2 µm) placed at 5 µm intervals. The movements of the molecules (D=5 $\mu m^2\ s^{-1}$) have apparent one dimensional random walk pattern (supplemental video S11) because the diameter of the tubes is smaller the diffraction-limited resolution of the optical microscopy.

Other important type of intracellular structures is microtubules and actin filaments forming cell cytoskeleton. Microtubules are built from α and β tubulin heterodimers usually forming 13 strands along microtubule (8 nm distance between the pairs of αβ tubulins) [8]. The actin stress fibres contain a bunch of parallel actin filaments, each build from actin monomers (5.5nm pitch step between the monomers). Both microtubules (Fig. S4E) and actin stress fibres can reach few tens of micrometers in length. Such structures pose a major problem for an object-based modelling because of the shear number of individual objects required for its representation. One approach of modelling the interactions between intracellular or membrane-bound objects and static linear structures is to use the physical coordinates of the potential binding sites on the linear structures, but do not represent these binding sites as individual objects. Another approach is to proportionally reduce the number of binding sites on the filaments to a few thousands, which allows to model single molecule interactions with the objects incorporated into the non-linear structures. The example Fig. S4F shows curved filaments made as the chains of immobile non-fluorescent objects placed at 8 nm distance from each other (from left to right). The random number generator was used during filament construction to make small changes in the y direction where the next object was placed relative to its predecessor. The cell was filled with free moving fluorescent objects (2 nM) which were allowed to bind to the binding sites on the filaments (R_{bind}=1×10^5 s^{-1}. ,R_{diss} =2 s^{-1}). After initial 5 minute run in darkness, required for the equalization, the ratio of bound / free floating molecules was equalized at ≈0.06. The 1000 frames long record (5 fps) was made under epi-illumination conditions (See part of this record on supplemental video S12). Fig. S4F, left panel shows a single image at the beginning of the record, and Fig. S4F, middle panel shows an averaged over 200 seconds image of the filamentous network created by all the fluorescent molecules temporarily bound to the filaments. An automatic spot detecting algorithm [3] was used to measure the x. y positions of the bound molecules with sub-pixel accuracy (~20nm) (Fig. S4F, right panel). This super resolution image shows some “false dots” placed between microtubules. This artefact was caused by the closely placed fluorescent molecules (distance < 0.5 µm) bound to the neighbouring microtubules at the same time. Supplemental video S13 shows a confocal scan made in similar conditions, but the filaments were allowed to curve in 3 dimensions and the R_{diss} was reduced to 0.02 s^{-1}, so that the majority of fluorescent objects were bound to the filaments.

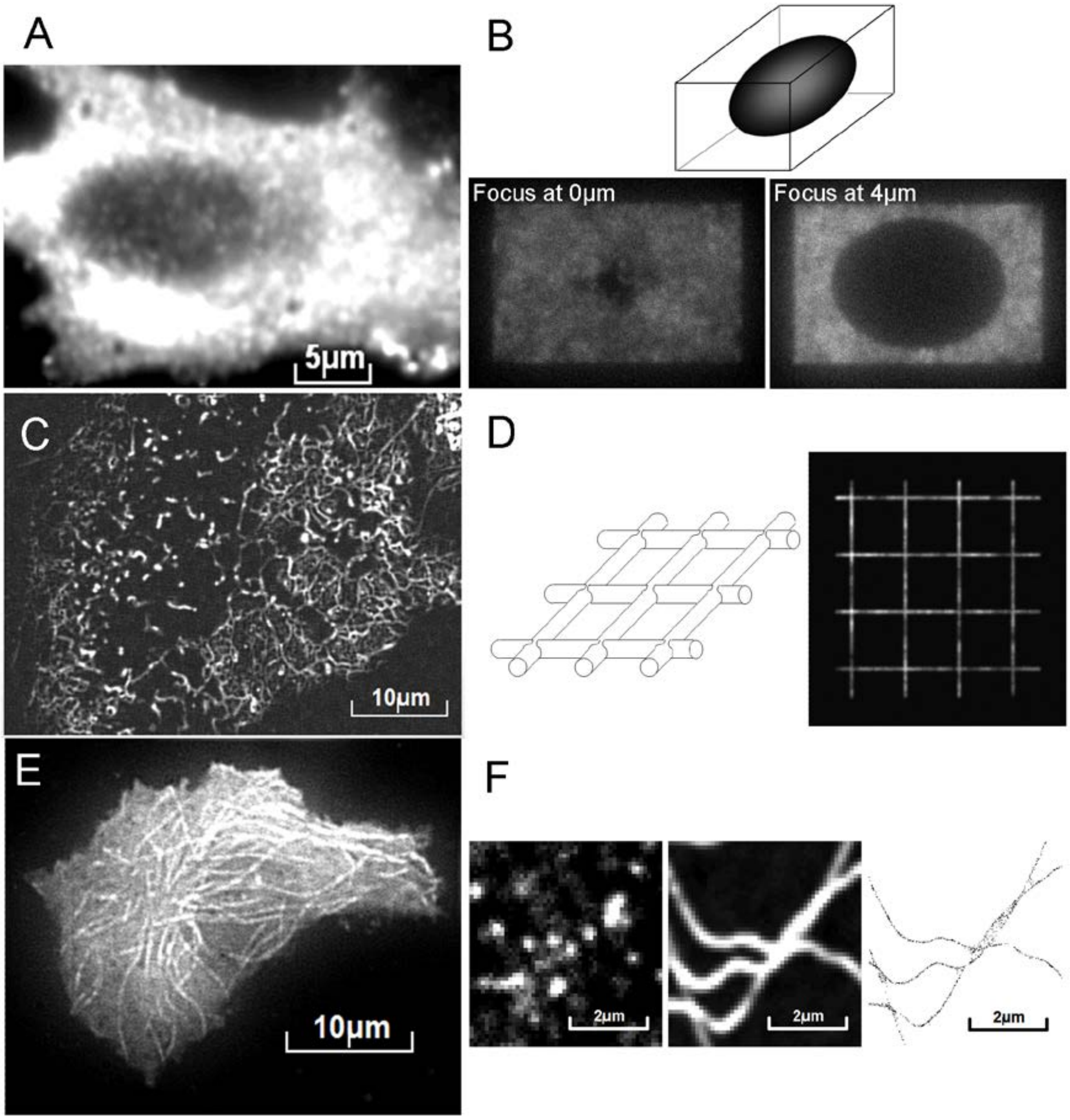

Figure S4. Intracellular structures. A. *Averaged over 3 seconds image of an endothelial cell transfected with a GFP-construct which does not penetrate nucleus (epi-illumination).* ***B.*** *Top panel - a diagram of a virtual cell with a nucleus represented as an ellipsoid impermeable for molecules moving in the cytoplasm. Left panel - a modelled cell with a nucleus imaged in epi-illumination mode. Concentration of fluorescent molecules was 5 nM, (D=5 $\mu m^2\ s^{-1}$). This image is an average of 300 model steps, total integration time 300 ms. Focal plane set at the bottom of the cell (z=0 µm). Cell sizes 20 × 10 × 8 µm (l × w × h). Right panel – the same "cell", but imaged at the focal plane lifted up to the half height of the cell (z=4 µm).* ***C.*** *TIRFM image of the potassium channels (KIR6.2-GFP) retained inside endothelium reticulum in HEK293 cell.* ***D.*** *Left - Tubular structures inside a cell can be represented as an interconnected network of tubules (Ø=200 nm) placed at regular intervals (5 µm). Molecules can move freely inside the tubules but unable to penetrate the tube walls. Right – A single image of a cell at the beginning of the model run made in the TIRFM mode. Tubes placed at the coverslip surface, focal plane at z=100 nm (See supplemental video S9).* ***E.*** *HEK293 cell transfected with tubulin-mRFP imaged in TIRFM mode.* ***F.*** *Bending microtubules were modelled as chains on immobile objects placed at 8 nm intervals. Intracellular fluorescent molecules were allowed to bind to microtubules (See text for details). Left panel – a single image shows few fluorescent molecules bound to microtubules lying at the focal plane (See supplemental video S12). Middle panel – An averaged over 1000 frames image showing all fluorescent molecules bound to the microtubules during the record. Right panel - The x, y positions of all the molecules detected in the record showed as dots.*

5. Simulating cell autofluorescence.

In some cases cell autofluorescence caused by intrinsic cellular biomolecules can affect the results of single molecule imaging experiments. This can be simulated either by introducing second class of cytoplasmic molecules or by introducing static structures containing fluorescent objects (See section S4 above). Usually cellular autofluorescent molecules are less bright compare to fluorophores attached to the protein of interest, but its abundance in a cell can affect the results of single molecule detection and tracking. For example: if we simulate single non-interacting membrane-embedded molecules freely diffusing on plasma membrane in a cell which also has dense network of tubular compartments (e.g., endoplasmic reticulum (Fig. S4C) or chains of mitochondria containing autofluorescent proteins) the results of tracking will be affected by cell autofluorescence. Fig. S5 shows the results of simulation done using the default conditions (See Section 2.6 in the main text). The concentration of free-diffusing autofluorescent proteins in the tubular network was 20 nM, while its fluorescence output was 4 times smaller of that of the molecules of interest (1500 versus 6000 photons s^{-1}). The results of tracking (Fig. S5C and D) show that the measured mobility was reduced to 0.255 $\mu m^2\ s^{-1}$, which is 15% smaller the set value of 0.3 $\mu m^2\ s^{-1}$ used in this simulation. If we remove the autofluorescence (Fig. S5CD, blue lines), the results of tracking will match the set value of 0.3 $\mu m^2\ s^{-1}$.

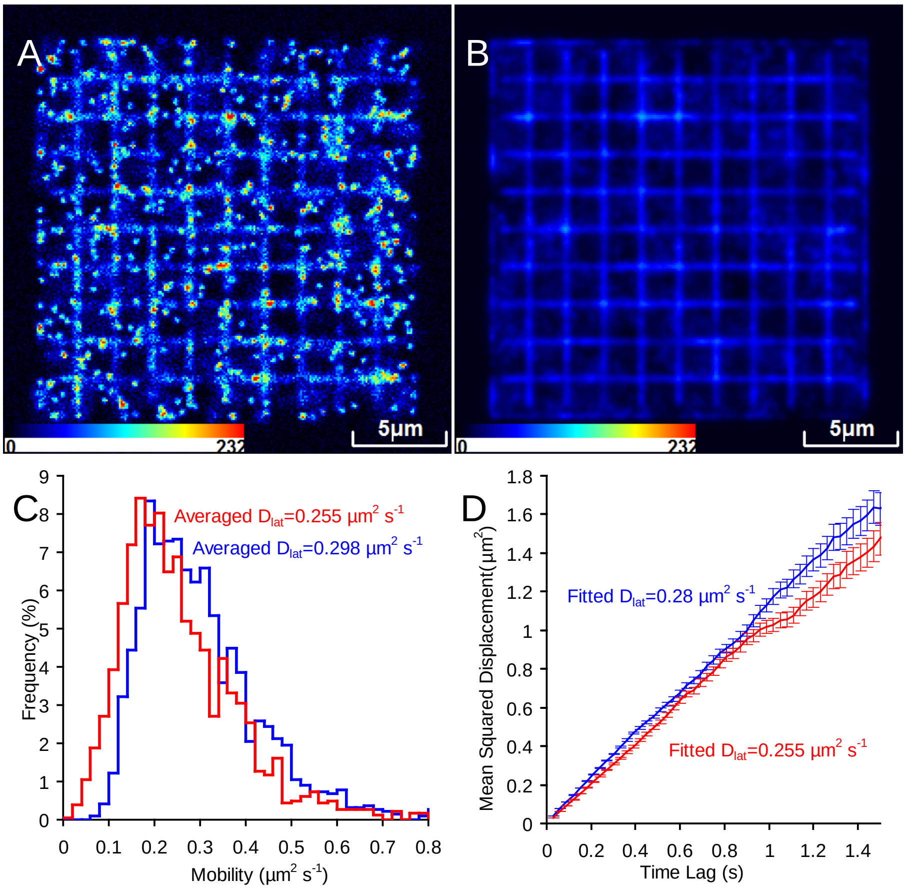

Fig. S5. Effect of cellular autofluorescence on the results single molecule tracking. *This is simulation of non-interacting membrane-based molecules free-diffusing on cell membrane imaged in Epi-illumination mode (See Section 2.6 in the main text for the conditions of modelling). The tubular network (Ø200 nm, distance between the tubes 2 µm, tubes placed horizontally at a height 0.5 µm, containing 20 nM of free-diffusing molecules emitting 1500 photons* s^{-1}, R_{bleach}*=0.5* s^{-1}*) was placed inside the cell to simulate cellular autofluorescence.* ***A.*** *First frame from the image sequence.* ***B.*** *Averaged image of the 200 frames sequence.* ***C.*** *Distribution of the measured* D_{lat} *of individual molecules with added source of autofluorescence (red line, 1895 objects) and without (blue line, 1808 objects).* ***D.*** *Averaged MSD -* $_{\Delta}t$ *(time lag) plots (±SEM) of molecules tracked in the presence (red line) and absence (blue line) of autofluorescence.*

6. Model interface.

The presented model can be downloaded from www.nimr.mrc.ac.uk/gmimpro as executable program GMcellModel.exe (Windows environment) and tested by independent investigator. The user can change all the parameters before and some during model run (See Fig. S6). These include: cell sizes; size and shape of nucleus; dimensions of tubular network; sizes and permeability level of membrane compartments; sizes and mobility level inside “lipid rafts”; interaction distance between reacting molecules; model time-step; number of molecules of each class (cytoplasmic A and B, membrane A, B, C, and static A); number and fraction of active fluorescent tags per molecule; diffusion rate; binding and dissociation rates; size of single fluorophore image and the defocusing coefficient; emission rates for green and red colour fluorescent tags; size of illuminating laser beam and iris diaphragm; TIRFM illuminating angle; number of model time-steps used to create single florescent image; Number of dark time-steps; camera noise; camera gain. The user can change some parameters counted above during model run (e.g., binding rates or bleaching rates) to simulate some changes in real experiments. The user can change the illumination mode, photobleach part of the specimen, switch lasers on and off or reset the model in the main menu of the program (Fig. S6E). It is also possible to change camera resolution (nm/pixel) and focus using interface in the main window.

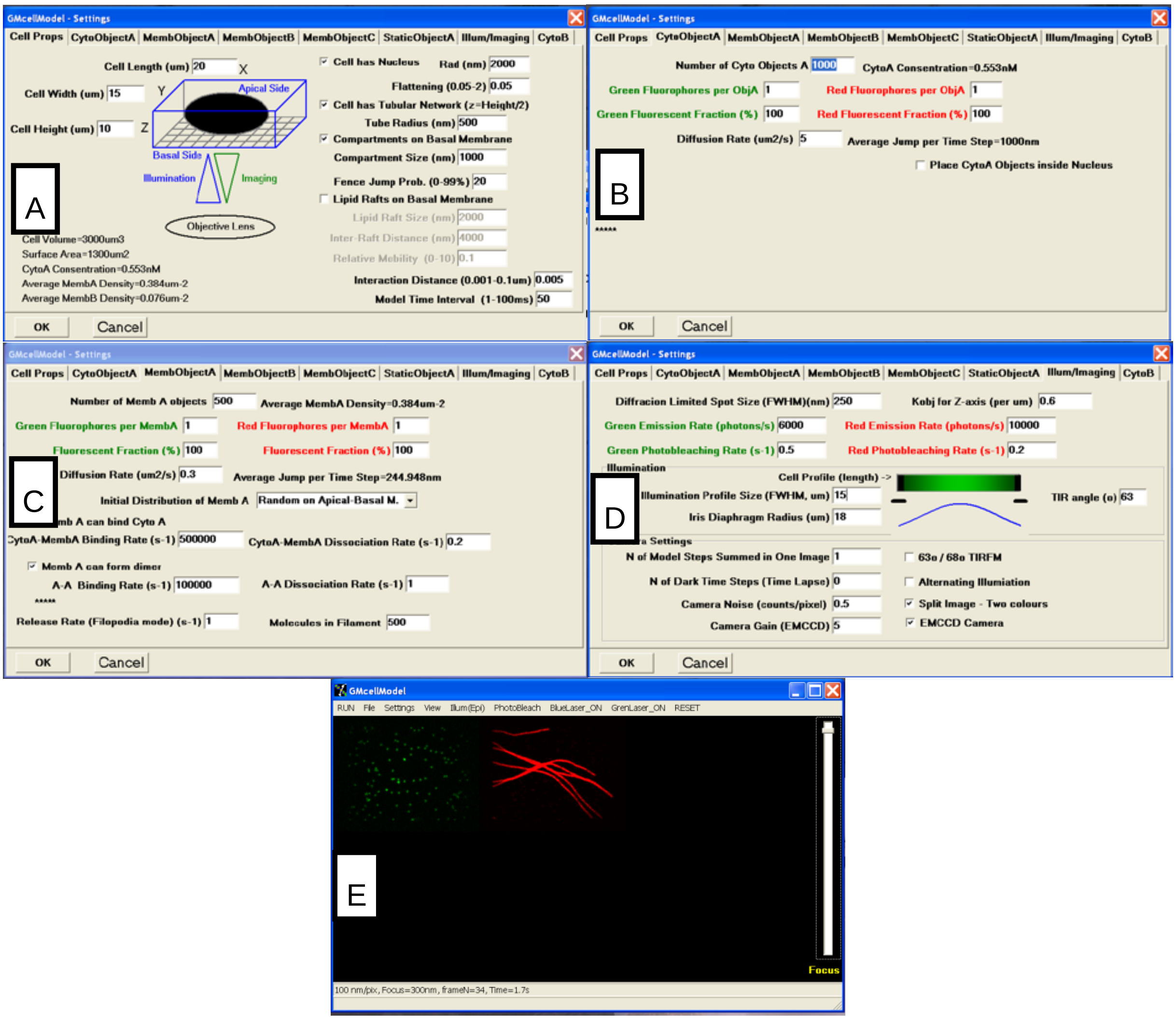

Fig. S6. The model interface. A. *Dialog window for simulated cell settings.* ***B.*** *Dialog window for simulated cytoplasmic molecules A.* ***C.*** *Dialog window for simulated membrane-based molecules A. A.* ***D.*** *Dialog window for adjusting imaging conditions.* ***E.*** *main program window showing main menu, focus track bar, and status bar at the bottom.*

7. Additional references:

[1] Nenasheva, T.A., Mashanov, G.I., Peckham, M. & Molloy, J.E. 2011 Imaging Individual Myosin Molecules Within Living Cells. In *Single Molecule Enzymology: Methods and Protocols* (pp. 123-142.
[2] Mashanov, G.I., Tacon, D., Knight, A.E., Peckham, M. & Molloy, J.E. 2003 Visualizing single molecules inside living cells using total internal reflection fluorescence microscopy. *Methods* **29**, 142-152. (doi:Pii s1046-2023(02)00305-5
10.1016/s1046-2023(02)00305-5).
[3] Mashanov, G.I. & Molloy, J.E. 2007 Automatic detection of single fluorophores in live cells. *Biophysical Journal* **92**, 2199-2211. (doi:10.1529/biophysj.106.081117).
[4] Hern, J.A., Baig, A.H., Mashanov, G.I., Birdsall, B., Corrie, J.E.T., Lazareno, S., Molloy, J.E. & Birdsall, N.J.M. 2010 Formation and dissociation of M-1 muscarinic receptor dimers seen by total internal reflection fluorescence imaging of single molecules. *Proceedings of the National Academy of Sciences of the United States of America* **107**, 2693-2698. (doi:10.1073/pnas.0907915107).
[5] Ulbrich, M.H. & Isacoff, E.Y. 2007 Subunit counting in membrane-bound proteins. *Nature Methods* **4**, 319-321. (doi:10.1038/nmeth1024).
[6] Kues, T., Peters, R. & Kubitscheck, U. 2001 Visualization and tracking of single protein molecules in the cell nucleus. *Biophysical Journal* **80**, 2954-2967.
[7] Olveczky, B.P. & Verkman, A.S. 1998 Monte Carlo analysis of obstructed diffusion in three dimensions: Application to molecular diffusion in organelles. *Biophysical Journal* **74**, 2722-2730.
[8] Leduc, C., Padberg-Gehle, K., Varga, V., Helbing, D., Diez, S. & Howard, J. 2012 Molecular crowding creates traffic jams of kinesin motors on microtubules. *Proceedings of the National Academy of Sciences of the United States of America* **109**, 6100-6105. (doi:10.1073/pnas.1107281109).
